# European consensus on pre-operative imaging in breast cancer developed jointly by EUSOBI, ESSO, ESP and ESTRO

**DOI:** 10.1007/s00330-026-12558-9

**Published:** 2026-06-16

**Authors:** Julia Camps-Herrero, Roberto Lo Gullo, Alexandra Athanasiou, Michael Fuchsjaeger, Fiona J. Gilbert, Youlia Kirova, Christiane Kuhl, Ritse Mann, Francesco Meani, Federica Pediconi, Ruud Pijnappel, Cristina Saura, Tibor Tot, Katja Pinker

**Affiliations:** 1 Breast Health, Ribera Salud Hospitals, Edificio Sorolla, Valencia, Spain; 2 Division of Breast Imaging, Department of Radiology, Columbia University, Vagelos College of Physicians and Surgeons, New York, NY USA; 3 Breast Imaging Department, MITERA Hospital, Maroussi, Greece; 4 Department of Radiology, Medical University of Graz, Graz, Austria; 5 Department of Radiology, University of Cambridge, Addenbrookes Hospital, Cambridge Biomedical Campus, Cambridge, United Kingdom; 6 Department of Radiation Oncology, Institut Curie, Paris, France; 7 Department of Diagnostic and Interventional Radiology, UKA, Hospital of RWTH Aachen University, Aachen, Germany; 8 Department of Imaging, Radboud University Medical Center, Nijmegen, The Netherlands; 9 Department of Radiology, The Netherlands Cancer Institute, Amsterdam, The Netherlands; 10 Department of Breast Surgery, Gruppo Ospedaliero Moncucco, Lugano, Switzerland; 11 Department of Radiological, Oncological and Pathological Sciences, Sapienza University of Rome, Rome, Italy; 12 Department of Radiology, University Medical Centre Utrecht, Utrecht, The Netherlands; 13 Medical Oncology Service, Vall d´Hebron University Hospital, Barcelona, Spain; 14 Pathology & Cytology Dalarna, County Hospital Falun, Falun, Sweden

**Keywords:** Breast neoplasms, Radiology, Consensus, Neoplasm staging

## Abstract

**Abstract:**

The current practice of pre-operative imaging in breast cancer is highly varied throughout Europe. Therefore, the European Society of Breast Imaging (EUSOBI) launched a call to all other European scientific societies involved in breast care to provide expert advice for pre-operative imaging in breast cancer and come to a common understanding of the types of evidence required for clinical practice guidelines for diagnostic tests. A panel comprising 13 experts (invited based on their level of expertise and representation in medical societies) voted on statements and questions encompassing all aspects of pre-operative staging. Consensus was reached in 67.4% of statements and questions, a majority in 28.3%, and no decision in 4.3%. Based on these findings, the panel developed a practical toolbox, based on currently available evidence and panel expertise, for the optimal breast cancer staging pathway considering all current imaging methods.

**Key Points:**

***Question*** *What is the role of breast MRI and other imaging methods in pre-operative staging in the light of current evidence?*

***Findings*** *Consensus was reached among panelists in 67.4% of statements and questions, a majority in 28.3%, and no decision in 4.3%.*

***Clinical relevance*** *A practical working toolbox for optimal breast cancer staging is provided based on currently available evidence and panel expertise.*

## Introduction

The current practice of pre-operative imaging of breast cancer is highly varied throughout Europe. Standard methods include conventional imaging such as mammography, digital breast tomosynthesis and breast ultrasound, and breast magnetic resonance imaging (MRI). Beyond these methods, contrast-enhanced mammography has also emerged as a valuable adjunct in the pre-operative imaging armamentarium for breast cancer staging, with current evidence supporting its use as an alternative to conventional mammography/digital breast tomosynthesis when MRI is not available or contraindicated, particularly for accurately delineating lesion extent and disease distribution [1, 2].

Breast MRI is the most accurate imaging method for depicting the extent of the tumor [3], but as shown in a recent survey by the European Society of Breast Imaging (EUSOBI) [4], its use is dependent on geographical location and hospital setting. In line with the EUSOBI guideline on breast MRI [5], the most common indications for pre-operative breast MRI include the presence of invasive lobular cancer, problem-solving in case of inconclusive findings on conventional imaging, screening of the contralateral breast in women with histological evidence of unilateral breast cancer, evaluation of the breasts in case of metastases of an unknown primary carcinoma, evaluation of therapy response in patients treated with neoadjuvant chemotherapy, exclusion of local recurrence after breast-conserving therapy, and screening of women with a breast cancer lifetime risk of 20% or more, including mutation carriers and women with dense breasts. A previous effort to develop a multidisciplinary guideline on the use of pre-operative breast MRI was made by the European Society of Breast Cancer Specialists (EUSOMA) [6]. In this guideline, the acceptable indications are: (1) patients newly diagnosed with an invasive lobular cancer (LoE-2a, DoR-B), (2) patients at high-risk for breast cancer (LoE-2b, DoR-B), (3) patients under 60 years of age with discrepancy in size > 1 cm between mammography and ultrasound with expected impact on treatment decision (LoE-2b, DoR-B), and (4) patients eligible for partial breast irradiation based on clinical breast examination and conventional imaging (LoE-3b, DoR-B). However, most of the evidence supporting this guideline came from when breast MRI was still regarded as an experimental modality and MRI-guided biopsy was not widely available. The diagnostic value of breast MRI has since been established, leading to its incorporation in routine clinical care. EUSOBI believes that the role of breast MRI, as well as that of other imaging methods in pre-operative staging, should be revised in the light of current evidence, also acknowledging the limitations of the validation methods used in the literature (e.g., conventional pathological sampling).

EUSOBI launched a call to other European scientific societies (European Society of Pathology (ESP), European Society for Medical Oncology (ESMO), European Society of Surgical Oncology (ESSO), and European Society of Radiation Oncology (ESTRO)) involved in breast care to provide expert advice for pre-operative imaging in breast cancer and come to a common understanding of the types of evidence required for clinical practice guidelines for diagnostic tests. An initial consensus meeting was held in Vienna, Austria, on January 9, 2020, to develop working packages (WPs) with consensus statements and questions encompassing all aspects of pre-operative staging with various imaging methods, which underwent three rounds of discussion and voting to reach consensus using a strict modified Delphi process (the last round of the questionnaire was sent in October 2023).

This manuscript provides a practical working toolbox, based on currently available evidence and panel expertise, for the optimal breast cancer staging pathway considering all current imaging methods.

## Findings

The methodology and full results of the modified Delphi process can be found in the supplementary material. After the first two rounds, 100% (13/13) of panelists voted on 123 statements/questions, with a consensus reached in 44.7%, a majority in 51.9%, and no decision in 3.3%. After the third round, 10 panelists voted on 123 statements/questions, with a consensus reached in 67.4%, a majority in 28.3%, and no decision in 4.3%. An overview describing each WP is shown in Table 1. All major statements derived from each WP are summarized in Table 2.

**Table 1:** Overview of the working packages

Working package (WP)	Description of WP
WP 1 (Supplementary Table 1)	Questions pertaining to the minimum technical requirements that ensure an optimal diagnosis in mammography, digital breast tomosynthesis, ultrasound, MRI, and contrast-enhanced mammography
WP 2 (Supplementary Table 2)	Questions pertaining to diagnostic breast imaging of women with operable breast cancer, with the intent to facilitate breast conservation
WP 3 (Supplementary Table 3)	Questions pertaining to diagnostic axillary imaging of women with operable breast cancer, with the intent to stage the axilla
WP 4 (Supplementary Table 4)	Questions pertaining to diagnostic tumor marking of the breast in women with operable breast cancer, with the intent to facilitate breast conservation
WP 5 (Supplementary Table 5)	Questions pertaining to intra-operative imaging of the specimen
WP 6 (Supplementary Table 6)	Questions pertaining to radiological-pathological correlation of breast cancer cases
WP 7 (Supplementary Table 7)	Questions pertaining to benchmark timelines for imaging studies during staging

**Table 2:** Toolbox on the clinical and technical requirements for the diagnosis of breast cancer

Working package	TOOLBOX
WP 1: Minimum technical requirements	• Bilateral mammography, or digital breast tomosynthesis plus synthetic mammography, with both craniocaudal and mediolateral oblique views, should always be performed before surgery. Spot-compression views and mammography after biopsy are not mandatory (required only after the placement of a marker). • Ultrasound of the whole affected breast should always be performed prior to surgery with a high-frequency probe and by a dedicated breast radiologist. • MRI when performed should include: –T1-weighted imaging before and after contrast administration, with or without fat suppression, with an in-plane and through-plane spatial resolution of ≤ 1 × 1 mm, and processing should include subtraction and maximum intensity projection images. –T2-weighted imaging with or without fat suppression and does not need to match the spatial resolution of the T1-weighted imaging. –Diffusion-weighted imaging with acquisition meeting EUSOBI consensus recommendations. • Contrast-enhanced mammography, when performed, should always consist of a low-energy and a recombined image with at least a craniocaudal and a mediolateral oblique view of both breasts, not only of the affected breast. The timing of the acquisition is important. Contrast-enhanced mammography is a valid alternative to mammography or DBT but not to MRI.
WP 2: Diagnostic breast imaging	• In accordance with the criteria issued by the Oxford Centre for Evidence-Based Medicine, the appropriate metric to rate the utility of diagnostic tests is diagnostic accuracy, and randomized trials are not needed to establish clinical use. The utility of MRI for pre-operative treatment corresponds to a level 1A evidence with a grade A recommendation. • Indications for breast MRI: Not all patients with breast cancer should undergo pre-operative breast MRI. In light of the current evidence, the following subgroups could benefit from pre-operative MRI: ◦ Patients with unifocal breast cancer and BI-RADS breast density A/B at diagnosis ◦ Patients with invasive breast cancer not otherwise specified and suspicion of multifocality/-centricity at conventional imaging or palpation. ◦ Patients with invasive lobular breast cancer ◦ Patients with either HER2-overexpressing breast cancer or triple-negative breast cancer ◦ Patients with unclear or discordant findings on mammography, digital breast tomosynthesis, and ultrasound with respect to lesion extent and presence of an extensive intraductal component ◦ Patients with a pre-operative diagnosis of high-grade ductal carcinoma in situ on stereotactic biopsy ◦ Patients who will undergo neoadjuvant therapy should have a breast MRI before and at the end of systemic treatment • Patients who cannot undergo MRI due to contraindications should be examined with contrast-enhanced mammography if feasible. • In patients with known cancer, additional findings identified by ultrasound or MRI require biopsy when the findings alter the treatment approach. • MRI findings that are made in addition to mammography should undergo work-up by second-look ultrasound; if the MRI findings do not have a correlate on second-look ultrasound, patients must undergo MRI-guided vacuum-assisted biopsy. • An institution should not offer pre-operative MRI when it is not able to arrange an MRI-guided biopsy inside or outside of the institution. • Additional MRI-detected suspicious lesions that potentially change the surgical plan should be properly bracketed. • After biopsy, two-view mammography should be obtained to document the position of the clip. • Patients with imaging exams from other hospitals should be thoroughly reviewed by in-house radiologists, and, if necessary, repeated or extended imaging is mandatory.
WP 3: Diagnostic axillary imaging	• Ultrasound is the method of choice to examine axillary lymph nodes and is mandatory in patients with palpable nodes and patients with a diagnosis of T1–T2 breast cancer. Fine needle aspiration/core biopsy of the most suspicious node should also be performed. • In case of neoadjuvant treatment with positive lymph nodes on fine needle aspiration or core biopsy (preferably the latter), lymph node marking before treatment should be done, and these lymph nodes should be removed after neoadjuvant treatment. The cut-off number of lymph nodes to be marked in the neoadjuvant treatment setting should be < 3. • There is no clear preference regarding the type of marker for marking lymph nodes. • Performance of CT or PET/CT in patients with confirmed metastatic lymph nodes is recommended.
WP 4: Diagnostic tumor marking	• Placement of markers should be performed in all non-palpable cancers, but it is not mandatory in all BI-RADS 4 or 5 lesions or in all palpable breast cancers. • Any type of marker can be used as long as it can be detected pre- and intra-operatively. • Placement of a guide wire: the guide wire should traverse the lesion center if the lesion is relatively spherical in shape. In case of a lesion with an irregular shape, there is no clear recommendation. • Biopsy and bracketing of additional multifocal or multicentric lesions should be discussed at the multidisciplinary team meeting and thoroughly planned beforehand. • The orientation protocol used in surgical specimens must be known by the radiologist and pathologist, and the removal of a localizing marker must be documented with a surgical specimen radiograph.
WP 5: Intra-operative imaging of the specimen	• Patients with bracketed complex lesions should be discussed (radiologist and surgeon) pre-operatively, and in patients with calcifications and/or pre-operatively placed markers, specimen radiography should be performed and evaluated by both, enabling real-time communication of the findings, especially if any of the markers is missing. • For better accuracy in special orientation, the specimen should be placed on its support by the surgeon. • If planned, intra-operative ultrasound should be performed both before beginning the surgical procedure in order to plan the resection, and afterward for gross evaluation of surgical margins. There is no recommendation regarding the guidance of targeted frozen sections of margins with multimodal imaging. • Intra-operative ultrasound of the specimen is only valid to locate the clip/marker, not for margin evaluation.
WP 6: Radiological-pathological correlation	• Thorough sampling of the specimen is recommended to document all radiologically evidenced abnormalities and also additional macroscopic abnormalities that were not evident at radiological examination. • Large-section histopathology has clear advantages over the traditional small block technique in this regard and is advocated.
WP 7: Benchmark timelines for imaging studies	• A comprehensive baseline assessment should be completed within 4 weeks of diagnosis. • Diagnosis and discussion of tailored treatment options should be completed within 7 working days. • Triple assessment (examination + imaging + biopsy) should be performed during the first clinical consultation without further delay. • Pre-operative MRI, when indicated, should be booked as early as the first consultation.

### Working package 1: Questions pertaining to the minimum technical requirements to ensure an optimal diagnosis in mammography, digital breast tomosynthesis, ultrasound, MRI, and contrast-enhanced mammography

#### Mammography and digital breast tomosynthesis

Consensus was reached that bilateral mammography, or digital breast tomosynthesis plus synthetic mammography, should always be performed before surgery, even when not performed in the diagnostic phase (90%) (this was a general statement that applies to all patients). All agreed that digital mammography should always consist of at least a craniocaudal and a mediolateral oblique view of each breast (100%). A majority voted against performing spot-compression views of the tumor area (60%). A majority also voted against repeating mammography after biopsy (70%), but if any marker has been placed, then a majority voted that mammography should be repeated (60%). While digital breast tomosynthesis alone was not considered a valid alternative to mammography (60%), digital breast tomosynthesis plus synthetic mammography was considered a valid alternative (80%). A consensus was also reached against performing digital breast tomosynthesis in one view only (70%).

#### Breast ultrasound

Consensus was achieved that ultrasound should be performed before surgery, even when not performed in the diagnostic phase (90%). There was a slight majority for performing an ultrasound in both breasts (60%). There was consensus on performing an ultrasound on the entire affected breast (80%), and correspondingly, that targeting the ultrasound to the area of the tumor and its surrounding tissue is insufficient (80%). Consensus was also achieved that ultrasound should be performed with a high-frequency linear transducer where possible (80%) and should not be performed with a curved lower frequency probe (70%). There was consensus that ultrasound should be performed by a dedicated breast radiologist (90%), and a majority voted against ultrasound being performed by a trained radiology technician (50%). Consensus was achieved that ultrasound images of the tumor should be captured in at least two planes (80%) and that four planes were unnecessary (80%). Consensus was achieved that ultrasound images of the normal breast should be documented (90%). There was unanimous agreement on the recommendation of color Doppler assessment for the tumor (100%). As for elastography, a simple majority agreed that it should be performed (50%). Panelists were not asked to vote on automated breast ultrasound (ABUS/AWUS) in the pre-operative staging setting. ABUS is not yet widely implemented for diagnostic staging across European institutions, and current evidence does not validate it as a replacement for manual high-frequency handheld axillary ultrasound, which remains the standard of care for clinical nodal assessment. ABUS may serve as a complementary tool for whole-breast lesion mapping or second-look evaluation in select centers, but it is not currently established or validated as a substitute for radiologist-performed manual axillary ultrasound in routine pre-operative breast cancer staging.

#### Breast MRI

A simple majority agreed that MRI should always be performed before surgery, even when not performed in the diagnostic phase (50%). This statement is similar to another statement in WP 2 stating that “all patients with breast cancer should undergo pre-operative breast MRI,” but the latter obtained a simple majority disagreement of 60% of the votes.

Regarding T1-weighted imaging, there was consensus that MRI should include T1-weighted imaging before and after contrast administration (80%). While the working package did not include statements regarding how many post-contrast sequences should be obtained, we note here that per established diagnostic breast MRI standards and current evidence, pre-operative staging should include at least two early post-contrast phases (to enable kinetic curve assessment and lesion conspicuity) and one delayed post-contrast sequence. The delayed phase is recommended to improve detection of lesions with progressive or delayed enhancement, including low-grade ductal carcinoma in situ and ductal carcinoma in situ components lacking strong early arterial uptake, which may be under-represented on abbreviated protocols.

There was consensus that the through-plane resolution should be ≤ 1 mm (70%), while a majority agreed that in-plane resolution should be ≤ 1 × 1 mm (50%). There was consensus that T1-weighted images could be obtained either with or without fat suppression (70%), and correspondingly, that fat suppression in all T1-weighted images is not necessary (70%). The panelists voted in consensus (80%) that subtraction images should always be created, and a majority (60%) also voted in favor of including maximum intensity projection images in the imaging protocol.

Regarding T2-weighted imaging, a majority considered it mandatory (60%), but there was no decision regarding the need for fat suppression or as to whether the T2-weighted imaging should match the in-plane or the through-plane spatial resolution of the T1-weighted acquisition.

Regarding diffusion-weighted imaging, a simple majority considered that it should be performed in all cases (50%), and consensus was reached that diffusion-weighted imaging acquisition should at least meet EUSOBI consensus recommendations (80%) [7]. The panel did not vote on specific slice-thickness thresholds for T2-weighted or DWI sequences. In clinical breast-MRI protocols, 4-mm sections for T2 and DWI remain standard to preserve signal-to-noise ratio and minimize distortion, motion sensitivity, and geometric warping in EPI-based diffusion. Thinner sections are not routinely recommended for pre-operative staging outside of targeted problem-solving or research protocols prioritizing non-EPI or readout-segmented acquisitions.

Of note, MRI technical parameters were posed to all stakeholders to ensure multidisciplinary alignment on minimum protocol standards required for clinically actionable pre-operative staging and correlation with specimen sampling. The role of surgeons and pathologists in this consensus is therefore to validate spatial fidelity and reporting completeness, not to direct selection of MRI physics parameters. Final protocol authority and optimization remain within the radiological profession.

#### Contrast-enhanced mammography

Similar to digital mammography, consensus was achieved that when contrast-enhanced mammography is performed, it should include at least a craniocaudal and a mediolateral oblique view of each breast (80%). A majority emphasized that contrast-enhanced mammography of the affected breast alone was insufficient (60%), and consensus was achieved regarding the importance of timing for contrast-enhanced mammography acquisitions, ideally performed between 2 and 8 min following contrast injection (70%). There was also consensus that each contrast-enhanced mammography view should consistently comprise a low-energy image and a recombined image (70%), and that evaluating recombined images only is inadequate (70%). Panelists agreed that contrast-enhanced mammography is a valid alternative to mammography or DBT (80%) but not to MRI (50%).

### WP 2: Questions pertaining to diagnostic breast imaging of women with operable breast cancer, with the intent to facilitate breast conservation

#### Sensitivity of breast MRI compared to conventional methods

There was consensus that MRI is more sensitive than mammography, DBT, or ultrasound (also known as conventional methods) to depict invasive breast cancer, regardless of the subtypes, as well as to identify and delineate the extent of ductal carcinoma in situ (DCIS) and the DCIS components of invasive breast cancer (90–100%). There was also consensus that the detectability of DCIS components of invasive breast cancer is not related to breast density (80%). There was unanimous agreement that MRI, mammography, DBT, and ultrasound are diagnostic methods for guiding treatment or for monitoring treatment effects (100%).

#### Criteria for diagnostic studies by the Oxford Centre for Evidence-Based Medicine

To dispel any misconceptions regarding the role and influence of MRI on surgical and oncological outcomes, panelists considered it important to recall and emphasize certain criteria regarding diagnostic tests.

In accordance with the criteria issued by the Oxford Centre for Evidence-Based Medicine, the appropriate metric to rate the utility of diagnostic tests is diagnostic accuracy, as agreed upon unanimously by the panelists (100%). Further, there was consensus that randomized trials are unnecessary to establish the clinical utility of diagnostic tests (80%). Additionally, there was consensus that validating diagnostic accuracy through cohort studies with robust reference standards (such as histology or thorough clinical follow-up) constitutes level 1 evidence (70%). According to a majority, when multiple level 1 studies produce consistent outcomes, level 1A evidence is attained (60%).

Based on these statements, a majority concurred that the evidence level regarding the utility of MRI for pre-operative treatment planning corresponds to level 1A evidence (60%). A higher percentage corresponding to consensus agreed that in the presence of multiple consistent level 1 studies regarding the diagnostic accuracy of MRI for pre-operative planning, the utilization of this method is recommended with a grade A (70%).

#### Indications for MRI

Regarding the indications for pre-operative MRI, a majority agreed that not all patients with breast cancer should undergo pre-operative MRI (60%); however, regarding which patients will particularly exhibit a benefit from pre-operative MRI, a majority acknowledged that there is currently not enough evidence to determine this subgroup (60%). In light of the current evidence, the following votes were obtained for who should undergo pre-operative MRI:

- A simple majority disagreed that patients with unifocal breast cancer and BI-RADS breast density A/B at diagnosis can be assessed with conventional imaging and do not need to undergo MRI (50%).
- All agreed that patients with invasive breast cancer not otherwise specified and suspicion of multifocality/-centricity at conventional imaging or palpation should undergo MRI (100%).
- There was consensus that patients with invasive lobular breast cancer should undergo MRI (90%).
- There was consensus that patients with either HER2-overexpressing breast cancer or triple-negative breast cancer should undergo MRI as a standard tool (90%).
- There was consensus that patients with unclear or discordant findings on mammography, digital breast tomosynthesis, and ultrasound with respect to lesion extent and the presence of extensive intraductal component (EIC) should undergo MRI (90%).
- All agreed that patients with a pre-operative diagnosis of high-grade DCIS on stereotactic biopsy should undergo MRI (100%).
- There was consensus that all patients who will undergo neoadjuvant therapy should have an MRI before and at the end of systemic treatment (90%).
- A slim majority admitted that at least 90% of patients with > 20–30% tumor volume at diagnosis (as diagnosed by conventional imaging methods or palpation) who will undergo neoadjuvant therapy with the intent to facilitate breast conservation should have MRI before and at the end of systemic treatment (50%).

Consensus was reached that patients who cannot undergo breast MRI due to contraindications such as severe claustrophobia, metallic implants (osteosynthesis material, pacemakers, implanted cardiac device, neurostimulators), or pregnancy should be examined before therapy with the imaging method that best depicts the lesion extent in their specific conditions (contrast-enhanced mammography if feasible) (90%). Pre-operative breast MRI is appropriate in lactating women when clinically indicated for loco-regional staging or problem-solving. Lactation is not a contraindication to MRI. To mitigate physiologic background parenchymal enhancement related to milk production, patients may be advised to breast-feed or pump immediately prior to the examination. Safety data support continued breastfeeding after gadolinium administration without mandatory interruption. Pre-operative MRI in lactating women should therefore be performed selectively, only when imaging findings may impact surgical or oncologic management [8].

#### Work-up of imaging findings

In patients with a known cancer, all accepted that additional findings identified by ultrasound or MRI require biopsy when the findings alter the treatment approach (change to more extensive surgery or change from breast conservation to mastectomy); exceptions can be made only when additional findings exhibit, by all measures, an identical imaging phenotype to the histologically proven index cancer (100%).

Regarding additional findings on MRI specifically, all acknowledged that MRI findings made in addition to mammography should undergo work-up by second-look ultrasound, and second-look ultrasound must be made by an experienced radiologist who is able to interpret the respective MRI finding and establish a correlation in terms of location, size, shape, and architecture (100%). All also acknowledged that MRI findings without a correlate on second-look ultrasound must undergo MRI-guided vacuum-assisted biopsy; in other words, access to MRI-guided vacuum-assisted biopsy must be ensured (100%). A majority agreed that since MRI is done to identify additional findings beyond mammography, DBT, and ultrasound, and since biopsy proof is required before one can act on additional MRI findings, an institution should not offer pre-operative MRI when it is not able to arrange an MRI-guided biopsy inside or outside of the institution (80%).

Additionally, all agreed that clear communication of imaging findings (mammography, DBT, ultrasound, MRI) into the operating room is essential; techniques to ensure such translation (lesion localization and bracketing) must be available for all imaging methods (100%).

#### Image-guided biopsies and bracketing

All agreed that additional MRI-detected suspicious lesions in the ipsilateral breast that can potentially change the surgical plan should be biopsied under image guidance for confirmation of the extent of disease and should be properly bracketed (100%).

Regarding clip placement, all agreed that if a lesion is small so that it might be partially or mostly removed by the biopsy, such that it will be difficult to re-identify the target area for pre-operative localization, a clip/marker must be placed (100%). Consensus was reached that if, during biopsy, a hematoma develops that might obscure the lesion during pre-operative localization, a clip must be placed (90%). Additionally, all agreed that in patients who undergo MRI-guided biopsy for a lesion visible by breast MRI alone, clips should be placed to facilitate pre-operative lesion localization under ultrasound or mammographic guidance (100%).

There was consensus that if a clip has been placed after biopsy, regardless of the type of imaging-guided procedure, two-view mammography (craniocaudal and mediolateral views) should be obtained to document the position of the clip (80%).

Almost all agreed that imaging exams from other hospitals should be thoroughly reviewed by in-house radiologists and imaging should be repeated or additional imaging should be performed if necessary (90%).

### WP 3: Questions pertaining to diagnostic axillary imaging of women with an operable breast cancer with the intent to stage the axilla

#### Role of breast ultrasound in lymph node staging and marking

There was consensus that ultrasound is the method of choice to examine axillary lymph nodes and that patients with T1–T2 breast cancer should undergo axillary ultrasound as well as fine needle aspiration or core biopsy of the most suspicious node if one is present (90%). A simple majority was achieved that in patients with breast cancer in medial quadrants (upper and lower inner quadrant), ultrasound should include the para-sternal region (50%). The Delphi questionnaire did not include voting items on supraclavicular or infraclavicular lymph node evaluation. Consequently, these higher-echelon regional nodal basins were not formally discussed or evaluated by the panel within the consensus process. Their assessment in clinical practice may be considered in selected staging or therapy-planning scenarios per institutional or national guidelines.

In cases of neoadjuvant treatment with positive lymph nodes on fine needle aspiration or core biopsy, there was consensus that lymph node marking before treatment should be done, and these lymph nodes should be removed after neoadjuvant treatment (80%). There was also consensus that the cut-off number of lymph nodes to be marked in the neoadjuvant treatment setting should be < 3 (70%). There was no decision regarding the type of marker preferred for marking lymph nodes (0–40%).

There was consensus that in the case of palpable lymph nodes on clinical examination, ultrasound should always be performed (90%). A majority agreed that in the case of confirmed metastatic lymph nodes, CT or PET/CT must always be performed before treatment (60%). There was consensus that in the case of neoadjuvant treatment with positive lymph nodes, core biopsy of the lymph node is mandatory to provide tumor tissue for adequate therapy-guiding tests (70%).

### WP 4: Questions pertaining to diagnostic tumor marking of the breast in women with an operable breast cancer with the intent to facilitate breast conservation

#### Pre-surgical localization

The majority agreed that in patients with BI-RADS 4 or 5 lesions, it is not mandatory to place a clip to facilitate pre-surgical localization (70%). Consensus was reached that, in all patients with a potential indication for later image-guided lesion localization, a marker that could be re-localized with ultrasound guidance is preferred (90%). Likewise, consensus was reached that any type of tumor localization at diagnosis can be used as long as pre-operative re-localization is feasible (80%). Finally, consensus was reached that pre-operative marking should be performed for all non-palpable breast cancers (90%), while a majority agreed that it should not be performed for all palpable breast cancers (60%).

#### Location of the marker

Concerning the placement of a guide wire, the consensus was that it should be positioned at the center of the target lesion if the lesion is relatively spherical in shape (80%). However, no decision was reached regarding its placement if the lesion exhibits an irregular shape.

#### Marking strategies

All agreed that all patients with additional lesions (multifocality, EIC) seen on MRI or any other imaging method should be discussed at the multidisciplinary team meeting in order to plan the pre-operative marking strategy (100%), and that direct interaction with surgeons is important to plan the localization and bracketing of lesions in order to help them understand where the guide wires were placed (100%). There was consensus that all additional lesions seen on MRI or any other imaging method should be pre-surgically biopsied and properly bracketed (80%). Further, there was consensus that it should be possible for any marker used for pre-operative staging to be re-localized intra-operatively (e.g., sonographic image, magnetic, radioactive, radiofrequency) (70%). All agreed that in patients with multicentric lesions and planned breast conservation, each lesion should be marked (100%).

#### Types of markers and marker evaluation

There was no decision regarding the preferred type of marker for the primary tumor (10–40%). All agreed that the orientation protocol used in surgical specimens must be known by the radiologist and pathologist (100%), and that the removal of a localizing marker must be documented with a surgical specimen radiograph (100%).

### WP 5: Questions pertaining to intra-operative imaging of the specimen

#### Role of intra-operative imaging

Regarding intra-operative imaging of the specimen, all agreed on the necessity for pre-operative discussion between the radiologist and surgeon for patients with complex lesions (i.e., non-unifocal) that have been marked (100%). Similarly, all agreed on performing specimen radiography for patients with calcifications and/or pre-operatively placed markers to confirm removal (100%), with real-time communication between the radiologist and surgeon during intra-operative X-ray evaluations (100%). Further, consensus was established that regardless of the X-ray source location for the specimen radiograph (Radiology department, Operating Room, or Pathology department), both the radiologist and surgeon must evaluate the specimen image and engage in real-time communication (90%) via phone, intercom, or secure messaging, enabling immediate clinical decisions before surgery concludes. Routine evaluation and feedback should occur within minutes of specimen acquisition, ideally before wound closure.

#### Specimen evaluation

All agreed that for better accuracy in spatial orientation, the specimen should be placed on its support by the surgeon (100%). Consensus was achieved that intra-operative ultrasound should be performed both before the surgical procedure in order to plan the resection, and afterward for gross evaluation of the surgical margins (80%). There was no decision regarding the guidance of targeted frozen section on margins with multimodal imaging.

Consensus was achieved that intra-operative ultrasound of the specimen is only valid to locate the clip/marker, not for margin evaluation (90%), and in the case of a missing marker in the specimen, as proven by X-ray or ultrasound, the surgeon should be informed immediately (100%).

### WP 6: Questions pertaining to radiological-pathological correlation of breast cancer cases

Regarding radiological-pathological correlation, consensus was unanimous (100%) regarding the following statements:

- Tumor size, disease extent, lesion focality, and heterogeneity are essential parameters that characterize breast carcinomas and should be reported as assessed with multimodality imaging methods pre-operatively and with detailed postoperative histopathological work-up of the specimen.
- The radiological assessment results of these parameters must be correlated with the results of histopathological assessment to reach definite interdisciplinary consensus diagnosis.
- Specimen radiographs (of the intact specimen, the sliced specimen, or both) facilitate this correlation by guiding the pathologist in sampling the tissue for histological examination and should be provided in all cases in which simple macroscopic examination may result in under-sampling of the specimen.

Almost all agreed that large-format histopathology has clear advantages over conventional sampling techniques in determining these parameters and should be preferably used (90%).

### WP 7: Questions pertaining to benchmark timelines for imaging studies during staging

Regarding benchmark timelines for imaging studies during staging, consensus was that every effort should be made to complete a comprehensive baseline assessment and commence treatment within 4 weeks from diagnosis (90%).

Regarding the timeline to provide a diagnosis, a majority agreed that patients should receive a diagnosis as soon as possible, acknowledging that some certification bodies require a diagnosis to be provided within 5 working days from the first visit, even if it means providing only partial information regarding the nature of the lesion (i.e., benign/malignant) (60%). However, most considered that a limited diagnosis, which does not allow discussing treatment details, is not beneficial for the patient (70%) and that waiting for a second consultation to discuss treatment options while being aware of the tumor diagnosis may increase the level of anxiety for the patient (80%). In this sense, most considered that a waiting time of 7 working days is reasonable to give a complete diagnosis and to discuss tailored treatment options (90%).

Almost all agreed that although some countries require allowing 24 h between informed consent and performing a breast biopsy, triple assessment (examination + imaging + biopsy) should be performed during the first clinical consultation without further delay to reduce waiting times (90%).

With the increasing use of pre-operative MRI, there are increasing treatment delays due to indications for further diagnostic assessment of suspicious findings on MRI. All agreed that, given the precise indications for pre-operative MRI, pre-operative MRI should be booked as early as the first consultation when it is indicated (100%).

## Discussion

Here, we provide a practical toolbox for breast cancer diagnosis and staging, based on the expert opinion of representatives from almost all European medical scientific societies involved in breast care (Table 2). Multiple areas of controversy were addressed within several specifically defined WPs representing specific clinical contexts.

WP 1 describes clear minimum technical requirements for all standard imaging modalities to ensure an optimal diagnosis. Meanwhile, WP 2 describes diagnostic breast imaging of women with operable breast cancer with the intent to facilitate breast conservation. In WP 2, the panel considered it important to recall and emphasize certain criteria with respect to how to rate the utility of diagnostic tests, aligning with the levels of evidence proposed by the Oxford Centre for Evidence-Based Medicine. The panel achieved consensus that in the presence of multiple consistent level 1 studies regarding the diagnostic accuracy of pre-operative MRI, the use of pre-operative MRI has a grade A recommendation [6]. Further, there was consensus that breast MRI is more sensitive than conventional imaging methods (mammography, digital breast tomosynthesis, or ultrasound) to depict invasive breast cancer and delineate the extent of DCIS. This aligns with existing literature indicating that MRI stands out as the most precise method for evaluating the extent of malignancy, exhibiting the greatest agreement in assessing the size of invasive ductal carcinoma, invasive lobular carcinoma, and DCIS compared to conventional imaging methods, along with having the highest negative predictive value [9–11].

Two similar questions regarding whether breast MRI should be recommended in all patients prior to breast cancer treatment were formulated in both WP 1 and WP 2. One elicited predominantly concurring opinions, while the other yielded predominantly conflicting viewpoints. Indeed, it is unsurprising that there is ongoing debate surrounding the appropriate use of breast MRI in newly diagnosed breast cancer patients [12]. Existing guidelines generally refrain from universally recommending systematic MRI for all breast cancer patients, considering it controversial or suitable only for specific circumstances, such as cases with invasive lobular cancer, cases requiring evaluation deep to the pectoral fascia, cases with discrepancies in disease extent between clinical examination and conventional diagnostic studies, and high-risk cases, among others [13–17]. Only the EUSOBI guidelines [5] advocate for pre-operative MRI in all breast cancer patients to screen the contralateral unaffected breast. The reason for such controversy stems from the fact that, although highly sensitive, MRI’s limited specificity potentially leads to false positives and an overestimation of disease, particularly if MRI findings are not biopsied before surgical excision. Due to this rationale, most of our panelists concurred that if an institution cannot arrange for an MRI-guided biopsy either internally or externally, it should not provide pre-operative MRI, as biopsy confirmation is necessary before acting on any additional MRI discoveries. Another reason for variability in responses between panelists may be related to consideration of cost and resource constraints. Panelists emphasized the importance of maintaining consistency in the utilization of imaging modalities rather than advocating for MRI in all patients. As a result, these issues have led to varied and inconsistent utilization of pre-operative MRI across different practice settings and among healthcare providers, as well as panelists who voted with majority agreement that there is not enough evidence to select a subgroup of women who will exhibit a benefit from the higher diagnostic accuracy associated with MRI. Lastly, while MRI can provide an improved assessment of breast cancer extent, this has not consistently translated into better clinical outcomes, such as reduced rates of re-excision or recurrence, due to a scarcity of well-designed studies examining these matters. Supporting and integrating this toolbox into ongoing and planned prospective research trials can enhance the level of evidence supporting current recommendations and provide future patients with increased access to diagnostic options grounded in evidence.

WP 3 describes the management of the axilla, emphasizing the leading role of ultrasound and the need to integrate this modality with interventional techniques in order to de-escalate unnecessary axillary surgery.

WP 4 and WP 5 describe diagnostic tumor marking and management of the surgical specimen. This part of the diagnostic process is as essential as the radiological diagnosis itself, because a brilliant diagnosis comes to nothing if its translation to the operating room is not performed in a careful and scrupulous way.

WP 6 describes radiological-pathological correlation in the era of breast MRI, highlighting a new mindset regarding the handling of the specimen, namely the need for thorough sampling of the surgical piece and the need for overcoming the limitations of the small block technique using large-section histology.

Finally, WP 7 describes benchmark timelines for imaging studies. Here, there was consensus that 7 working days is a valid timeline for giving a complete diagnosis and discussing the different treatment options. This entails that during the first clinical consultation, a triple assessment should be performed, and pre-operative MRI should be booked without further delay. This baseline assessment should be completed within 4 weeks in order to start treatment.

The program’s limitations highlight potential unintentional biases in guidance statements stemming from specialties less acquainted with surgical, pathology, and diagnostic techniques, as evidenced by their abstention from voting.

## Conclusion

This consensus provides clinical guidance recommendations and a practical toolbox to help with the clinical and technical requirements associated with the diagnosis of breast cancer. This guidance could assist clinicians and patients in understanding and standardizing the optimal breast cancer staging pathway with current imaging methods. Of note, variability in real-world implementation reflects appropriate adaptation to local resources and clinical settings, and is not interpreted as a deviation from the need for evidence-based diagnostic guidance.

## Supplementary information

ELECTRONIC SUPPLEMENTARY MATERIAL

## Abbreviations

DBT: Digital breast tomosynthesis
DCIS: Ductal carcinoma in situ
ESMO: European Society for Medical Oncology
EUSOMA: European Society of Breast Cancer Specialists
EUSOBI: European Society of Breast Imaging
ESP: European Society of Pathology
ESTRO: European Society of Radiation Oncology
ESSO: European Society of Surgical Oncology
EIC: Extensive intraductal component
WPs: Working packages
