# Supplementary material for: European consensus on pre-operative imaging in breast cancer developed jointly by EUSOBI, ESSO, ESP and ESTRO
Source: Eur Radiol. 2026 Jun 16;36(10):7806–16. doi: 10.1007/s00330-026-12558-9 (PMC13574875; doi:10.1007/s00330-026-12558-9)
Supplement: Supplementary file 1 [file 330_2026_12558_MOESM1_ESM.docx]

**SUPPLEMENTARY MATERIAL**

Contents

**DATA COLLECTION**	2

**Supplemental Table 1. WP 1: Questions pertaining to the minimum technical requirements that ensure an optimal diagnosis in mammography, digital breast tomosynthesis, ultrasound, MRI, and contrast-enhanced mammography.**	4

**Supplemental Table 2. WP 2: Questions pertaining to diagnostic breast imaging of women with an operable breast cancer with the intent to facilitate breast conservation.**	6

**Supplemental Table 3. WP 3: Questions pertaining to diagnostic axillary imaging of women with an operable breast cancer with the intent to stage the axilla.**	9

**Supplemental Table 4. WP 4: Questions pertaining to diagnostic tumor marking of the breast in women with an operable breast cancer with the intent to facilitate breast conservation.**	10

**Supplemental Table 5. WP 5: Questions pertaining to intraoperative imaging of the specimen.**	11

**Supplemental Table 6. WP 6: Questions pertaining to the radiological-pathological correlation of breast cancer cases.**	12

**Supplemental Table 7. WP 7: Questions pertaining to benchmark timelines for imaging studies during staging.**	13

# **DATA COLLECTION**

**Steering Committee and Voting Panel**

The steering committee (SC) comprised 11 members with expertise in breast cancer diagnosis and treatment across seven European countries and the United States. Represented specialties included breast imaging, surgery, radiation oncology, pathology, and medical oncology. Members were invited based on their level of expertise and representation in medical scientific societies. The SC determined the clinical statements and questions and subsequently the seven working packages (WPs) based on all stages of the patient journey during the diagnostic staging process. This methodology allowed the panelists at the final consensus meeting to focus on, and vote on, multiple areas of controversy. A systematic search of the literature was not performed.

The program was endorsed by the following European societies: ESSO, ESTRO, EUSOBI, and ESP. Each society delegated a representative with specific expertise to join the multidisciplinary voting panel. One representative from EUROPA DONNA joined the panel but could no longer commit in the ensuing months. The final panel (including the SC members) comprised 13 representatives from seven European countries and the United States, and covered six specialties (surgery, radiology, radiation oncology, gynecology, medical oncology, and pathology). The medical oncologist was not a representative of ESMO and voted on a personal basis.

Final panel:

1. Julia Camps-Herrero
2. Alexandra Athanasiou
3. Michael Fuchsjaeger
4. Fiona Gilbert
5. Youlia Kirova
6. Christiane Kuhl
7. Ritse Mann
8. Francesco Meani
9. Federica Pediconi
10. Ruud Pinappel
11. Cristina Saura
12. Tibor Tot
13. Katja Pinker

**Meeting Procedures**

A modified Delphi process was conducted, including preparation, consensus, and reporting:

1. An initial face-to-face consensus meeting with the SC took place on January 9, 2020. The working methodology was explained to the whole panel and the current evidence reviewed. WPs were defined and assigned to be developed by members of the SC. Final WPs with statements and questions regarding areas of controversy were sent to panelists twice between January 9, 2020 and January 20, 2021. Of the 13 panelists, 84.6% (11/13) answered the first round of questions.
2. During the second consensus meeting of January 20, 2021, which occurred virtually due to the COVID-19 pandemic, only the statements and questions that did not reach any consensus were discussed and then sent for a second anonymous review to all panelists via email. Of the 13 voting panelists, 53·6% (7/13) answered the second round of questions. After consolidation of all input, 100% (13/13) provided further answers and agreed to the semi-final version.
3. In September 11 2023, the same statements and questions were sent via email to the 13 panelists along with an update in the scientific evidence on radiologic breast cancer staging to overcome limitations of the time elapsed since the first meeting. Of the 13 panelists, 76.9% (10/13) answered the third round of questions. These answers constitute the final consensus on which the toolbox recommendations were built.

**Voting**

Voting was carried out anonymously. For the initial two rounds of voting, consensus was achieved if ≥ 75% of all panelists concurred on a specific statement or question, following the commonly cited definition of consensus for Delphi studies [1]. Majority was defined as 51–74% of the votes. ≤ 50% agreement resulted in no decision. Cases where votes were equally divided between two options or when the majority of votes were abstentions were deemed "no decision.” For the third round of voting, given that 76.9% (10/13) panelists voted, consensus was achieved if ≥ 70% of the 10 panelists who voted concurred on a statement or question. Majority was defined as 50%–69% of the votes. ≤ 49% agreement resulted in no decision.

1. Diamond IR, Grant RC, Feldman BM, Pencharz PB, Ling SC, Moore AM, et al (2014) Defining consensus: a systematic review recommends methodologic criteria for reporting of Delphi studies. J Clin Epidemiol 67:401–409

# **Supplemental Table 1. WP 1: Questions pertaining to the minimum technical requirements that ensure an optimal diagnosis in mammography, digital breast tomosynthesis, ultrasound, MRI, and contrast-enhanced mammography.**

| Statements | No. of responses | Percentage votes | | | Voting result |
| --- | --- | --- | --- | --- | --- |
|  |  | **Yes (n)** | **No (n)** | **Abstain (n)** |  |
| Bilateral mammography (or DBT+ synthetic mammography) should always be available. before surgery, even when not performed in the diagnostic phase | 10 | 90% (9) | 10% (1) | 0% (0) | Yes (Consensus) |
| Digital Mammography should always consist of at least a CC and an MLO view of each breast | 10 | 100% (10) | 0% (0) | 0% (0) | Yes (Consensus) |
| Spot-compression views of the tumor area are always required | 10 | 40% (4) | 60% (6) | 0% (0) | No (Majority) |
| Mammography should be repeated after biopsy | 10 | 30% (3) | 70% (7) | 9% (1) | No (Majority) |
| Mammography should be repeated after placement of any marker | 10 | 60% (6) | 40% (4) | 0% (0) | Yes (Majority) |
| DBT alone is a valid alternative for mammography | 10 | 20% (2) | 60% (6) | 20% (2) | No (Majority) |
| DBT+ synthetic mammography is a valid alternative for mammography | 10 | 80% (8) | 10% (1) | 10% (1) | Yes (Consensus) |
| DBT may be performed in one view (MLO) only | 10 | 20% (2) | 70% (7) | 10% (1) | No (Consensus) |
| Ultrasound should always be performed prior to surgery, even when not performed in the diagnostic phase | 10 | 90% (9) | 10% (1) | 0% (0) | Yes (Consensus) |
| Ultrasound should always be performed of both breasts | 10 | 60% (6) | 30% (3) | 10% (1) | Yes (Majority) |
| Ultrasound should always be performed of the whole affected breast | 10 | 80% (8) | 20% (2) | 0% (0) | Yes (Consensus) |
| Targeted ultrasound of the tumor and its surrounding is sufficient | 10 | 20% (2) | 80% (8) | 0% (0) | No (Consensus) |
| Ultrasound should be performed with a high frequency linear transducer when possible | 10 | 90% (9) | 0% (0) | 10% (1) | Yes (Consensus) |
| Ultrasound can be performed with a curved lower frequency probe in the majority of cases | 10 | 10% (1) | 70% (7) | 20% (2) | No (Consensus) |
| Ultrasound should be performed by a dedicated breast radiologist | 10 | 90% (9) | 10% (1) | 0% (0) | Yes (Consensus) |
| Ultrasound may be performed by a trained radiology technician | 10 | 40% (4) | 50% (5) | 9% (1) | No (Consensus) |
| Ultrasound images of the tumor should be captured in at least 2 planes | 10 | 80% (8) | 0% (0) | 20% (2) | Yes (Consensus) |
| Ultrasound images of the tumor should be captured in at least 4 planes | 10 | 0% (0) | 80% (8) | 20% (2) | No (Consensus) |
| Ultrasound images of the normal breast should be documented | 10 | 80% (8) | 20% (2) | 0% (0) | Yes (Consensus) |
| Color doppler assessment of the tumor is suggested | 10 | 100% (8) | 0% (0) | 0% (0) | Yes (Consensus) |
| Elastography assessment of the tumor is suggested | 10 | 50% (5) | 30% (3) | 20% (2) | Yes (Majority) |
| Contrast enhanced MRI should always be performed prior to surgery, even when not performed in the diagnostic phase | 10 | 50% (5) | 30% (3) | 20% (2) | Yes (Majority) |
| MRI should include T1 weighted images before and after contrast administration | 10 | 80% (9) | 0% (0) | 20% (2) | Yes (Consensus) |
| In plane resolution should be 1 x 1 mm or below | 10 | 50% (5) | 20% (2) | 30% (3) | Yes (Majority) |
| Through plane resolution should be 1mm or below | 10 | 70% (7) | 0% (0) | 30% (3) | Yes (Consensus) |
| T1 weighted images can be obtained with or without fat-suppression (any kind) | 10 | 70% (7) | 0% (0) | 30% (3) | Yes (Consensus) |
| T1 weighted images require fat-suppression | 10 | 0% (0) | 70% (7) | 30% (3) | No (Consensus) |
| Subtraction images should always be created | 10 | 80% (9) | 0% (0) | 20% (2) | Yes (Consensus) |
| Maximum intensity projections should always be created | 10 | 60% (6) | 0% (0) | 40% (4) | Yes (Majority) |
| T2 weighted images should always be obtained | 10 | 60% (6) | 10% (1) | 30% (3) | Yes (Majority) |
| T2 weighted images can be obtained with or without fat-suppression (any kind) | 10 | 70% (7) | 0% (0) | 30% (3) | Yes (Consensus) |
| T2 weighted images require fat-suppression | 10 | 30% (3) | 40% (4) | 30% (3) | No Decision |
| T2 weighted images should match the spatial resolution of the T1 weighted acquisition in plane | 10 | 50% (5) | 0% (0) | 50% (5) | No Decision |
| T2 weighted images should match the spatial resolution of the T1 weighted images through plane | 10 | 20% (2) | 30% (3) | 50% (5) | No Decision |
| DWI weighted sequences should always be obtained | 10 | 50% (5) | 20% (2) | 30% (3) | Yes (Majority) |
| DWI acquisitions should at least meet the current EUSOBI consensus statement | 10 | 80% (9) | 0% (0) | 20% (2) | Yes (Consensus) |
| CESM should always consist of at least a CC and a MLO view of each breast | 10 | 80% (9) | 0% (0) | 20% (2) | Yes (Consensus) |
| CESM of the affected breast is sufficient | 10 | 10% (1) | 60% (6) | 30% (3) | No (Majority) |
| Timing of CESM acquisitions is irrelevant | 10 | 0% (0) | 70% (7) | 30% (3) | No (Consensus) |
| Each CESM view should always consist of a low-energy image and a recombined image | 10 | 70% (7) | 0% (0) | 30% (3) | Yes (Consensus) |
| CESM images can be evaluated using only the recombined image | 10 | 0% (0) | 70% (7) | 30% (3) | No (Consensus) |
| CESM is a valid alternative for mammography or DBT | 10 | 80% (9) | 0% (0) | 20% (2) | Yes (Consensus) |
| CESM is a valid alternative for MRI | 10 | 30% (3) | 50% (5) | 20% (2) | No (Majority) |

# **Supplemental Table 2. WP 2: Questions pertaining to diagnostic breast imaging of women with an operable breast cancer with the intent to facilitate breast conservation.**

| Statements | No. of responses | Percentage votes | | | Voting result |
| --- | --- | --- | --- | --- | --- |
|  |  | **Yes (n)** | **No (n)** | **Abstain (n)** |  |
| Breast MRI is more sensitive than mammography, DBT or ultrasound to depict invasive breast cancer | 10 | 100% (10) | 0% (0) | 0% (0) | Yes (Consensus) |
| For lobular invasive cancer MRI is more sensitive than mammography, DBT and ultrasound | 10 | 90% (9) | 10% (1) | 0% (0) | Yes (Consensus) |
| Breast MRI is more sensitive than mammography, DBT or ultrasound for invasive cancer regardless of invasive cancer type (it is more sensitive for NST-cancers, lobular cancers, primary or recurrent cancers | 10 | 100% (10) | 0% (0) | 0% (0) | Yes (Consensus) |
| Breast MRI is more sensitive than mammography, tomosynthesis or ultrasound to identify and delineate extent of DCIS | 10 | 90% (9) | 0% (0) | 10% (1) | Yes (Consensus) |
| Breast MRI is more sensitive than mammography, tomosynthesis or ultrasound to depict DCIS components of invasive breast cancer | 10 | 90% (9) | 0% (0) | 10% (1) | Yes (Consensus) |
| Detectability of DCIS components of invasive breast cancer is not related to breast density | 10 | 80% (9) | 0% (0) | 20% (2) | Yes (Consensus) |
| The value of breast MRI regarding the additional detection of DCIS or DCIS components is independent of mammographic breast density | 10 | 80% (9) | 0% (0) | 20% (2) | Yes (Consensus) |
| Breast MRI, mammography, DBT and ultrasound are diagnostic methods that are used to guide treatment, or monitor treatment effects | 10 | 100% (10) | 0% (0) | 0% (0) | Yes (Consensus) |
| In accordance with the Center of Evidence Based Medicine, the appropriate metric to rate the utility of diagnostic tests is diagnostic accuracy | 10 | 100% (10) | 0% (0) | 0% (0) | Yes (Consensus) |
| In accordance with the Center of Evidence Based Medicine, randomized trials are not needed to establish the clinical use of diagnostic tests; rather, intra-individual comparison studies that determine the respective diagnostic accuracies, with blinded interpretation of diagnostic tests, and validation by a sound reference standard (histology or adequate clinical follow-up), correspond to an adequate study design | 10 | 80% (9) | 0% (0) | 20% (2) | Yes (Consensus) |
| In accordance with the Center of Evidence Based Medicine, validating cohort studies on the respective diagnostic accuracy with sound reference standard are considered level-1 evidence | 10 | 70% (7) | 0% (0) | 30% (3) | Yes (Consensus) |
| In accordance with the Center of Evidence Based Medicine, level-1A evidence for diagnostic studies is achieved when several level-1 studies (cross-sectional intra-individual comparison studies with valid reference standard) yield concordant results (systematic review of level 1 studies with homogeneity). | 10 | 60% (6) | 0% (0) | 40% (4) | Yes (Majority) |
| In accordance with the criteria issued by the Center of Evidence Based Medicine, the level of evidence regarding the utility of MRI for preoperative treatment planning corresponds to a level 1A | 10 | 60% (6) | 10% (1) | 30% (3) | Yes (Majority) |
| With several consistent level-1 studies regarding the diagnostic accuracy of breast MRI for preoperative planning, use of this method is recommended with a grade A | 10 | 70% (7) | 10% (1) | 20% (2) | Yes (Consensus) |
| All patients with a breast cancer should undergo pre-operative breast MRI. | 10 | 40% (4) | 60% (6) | 0% (0) | No (Majority) |
| There is insufficient evidence to select a subgroup of women who will not exhibit a benefit from the higher diagnostic accuracy associated with MRI | 10 | 60% (6) | 20% (2) | 20% (2) | Yes (Majority) |
| Patients with unifocal breast cancer and BI-RADS breast density A/B at diagnosis can be assessed with conventional imaging and do not need MRI. | 10 | 20% (2) | 50% (6) | 30% (3) | No (Majority) |
| Patients with invasive breast cancer NOS and suspicion of multifocality/-centricity in conventional imaging or palpation should undergo staging MRI | 10 | 100% (10) | 0% (0) | 0% (0) | Yes (Consensus) |
| Patients with invasive lobular breast cancer should undergo staging MRI | 10 | 90% (9) | 10% (1) | 0% (0) | Yes (Consensus) |
| Patients with either HER2 overexpressing tumors or TNBC should undergo MRI imaging as a standard tool | 10 | 90% (9) | 10% (1) | 0% (0) | Yes (Consensus) |
| Patients with unclear or discordant findings in mammography/DBT/US with respect to lesion extent and presence of EIC should undergo staging MRI | 10 | 90% (9) | 10% (1) | 0% (0) | Yes (Consensus) |
| All patients that will undergo neoadjuvant therapy should have a breast MRI before and at end of systemic treatment | 10 | 90% (9) | 0% (0) | 10% (1) | Yes (Consensus) |
| At least 90% of patients with >20-30% tumor volume at diagnosis (as diagnosed by conventional imaging mammography, digital breast tomosynthesis and/or ultrasound or palpation) that will undergo neoadjuvant therapy with the intent to facilitate breast conservation should have a breast MRI before and at end of systemic treatment | 10 | 50% (5) | 20% (2) | 30% (3) | Yes (Majority) |
| Patients with pre-operative diagnosis of High-Grade DCIS on stereotactic biopsy should undergo MRI | 10 | 100% (10) | 0% (0) | 0% (0) | Yes (Consensus) |
| Patients that cannot undergo MRI assessment of disease due to contraindications such as severe claustrophobia, metallic implants (osteosynthesis material, pacemakers, ICD, neurostimulators) or pregnancy should be examined before therapy with the imaging modality that best depicts the lesion extent in their specific conditions (Contrast-enhanced mammography if feasible) | 10 | 90% (10) | 0% (0) | 10% (1) | Yes (Consensus) |
| In a BI-RADS-6 patient, additional findings identified by ultrasound or MRI require biopsy when the findings alter the treatment approach (change to more extensive surgery, or change from breast conservation to mastectomy); exceptions can be made only when additional findings exhibit, by all measures, an identical imaging phenotype to the histologically proven index cancer | 10 | 100% (10) | 0% (0) | 0% (0) | Yes (Consensus) |
| MR imaging findings that are made in addition to mammography should undergo work up by second look ultrasound; second look ultrasound must be made by an experienced radiologist who is able to interpret the respective MRI finding and establishes a correlation in terms of location, size, shape, and architecture | 10 | 100% (11) | 0% (0) | 0% (0) | Yes (Consensus) |
| MRI findings that do not have a correlate on second-look US must undergo MR-guided vacuum-assisted biopsy; in other words: Access to MR-guided vacuum-assisted biopsy must be ensured | 10 | 100% (10) | 0% (0) | 0% (0) | Yes (Consensus) |
| Since MRI is done to identify additional findings beyond mammography, DBT and ultrasound, and since biopsy proof is required before one can act on additional MRI findings, an institution should not offer preoperative MRI when it is not able to arrange an MR guided biopsy inside or outside of the institution | 10 | 80% (9) | 0% (0) | 20% (2) | Yes (Consensus) |
| Clear communication of imaging findings (mammography, DBT, ultrasound, MRI) into the operating room is essential; techniques to ensure such translation (lesion localization and bracketing) must be available for all imaging methods (mammography, DBT, US, and MRI guidance). | 10 | 100% (10) | 0% (0) | 0% (0) | Yes (Consensus) |
| Additional MRI-detected suspicious lesions that potentially change the surgical plan in the ipsilateral breast should be biopsied under image guidance for confirmation of extent of disease and properly bracketed | 10 | 100% (10) | 0% (0) | 0% (0) | Yes (Consensus) |
| If a lesion is small so that it might be partially or mostly removed by the biopsy, such that it will be difficult to re-identify the target area for pre-operative localization, a clip/marker must be placed. | 10 | 100% (10) | 0% (0) | 0% (0) | Yes (Consensus) |
| If, during biopsy, a hematoma develops that might interfere with target detectability that might obscure the lesion during pre-operative localization, a clip must be placed | 10 | 90% (10) | 10% (1) | 0% (0) | Yes (Consensus) |
| In a patient who undergoes MR-guided biopsy for a lesion visible by breast MRI alone, clips should be placed to facilitate pre-operative lesion localization under ultrasound or mammographic guidance | 10 | 100% (10) | 0% (0) | 0% (0) | Yes (Consensus) |
| If, after biopsy, regardless of type of procedure (mammography-guided, DBT-guided, US-guided or MR-guided), a clip has been placed, two-view mammography (mediolateral and cc views) should be obtained to document the position of the clip | 10 | 80% (7) | 10% (1) | 10% (1) | Yes (Consensus) |
| Patients with imaging exams from other hospitals should be thoroughly reviewed by in-house radiologists and if necessary repeated or extended imaging is mandatory | 10 | 100% (10) | 0% (0) | 0% (0) | Yes (Consensus) |

# **Supplemental Table 3. WP 3: Questions pertaining to diagnostic axillary imaging of women with an operable breast cancer with the intent to stage the axilla.**

| Statements | No. of responses | Percentage votes | | | Voting result |
| --- | --- | --- | --- | --- | --- |
|  |  | **Yes (n)** | **No (n)** | **Abstain (n)** |  |
| Ultrasound is the method of choice to examine axillary lymph nodes | 10 | 90% (10) | 10% (1) | 0% (0) | Yes (Consensus) |
| Patients with a diagnosis of a T1-T2 breast cancer should undergo axillary ultrasound | 10 | 90% (10) | 10% (1) | 0% (0) | Yes (Consensus) |
| In patients with breast cancer in medial quadrants (upper and lower inner quadrant), ultrasound should also include the para-sternal region | 10 | 50% (5) | 30% (3) | 20% (2) | Yes (Majority) |
| Patients with suspicious lymph nodes should undergo FNA/Core biopsy of the most suspicious node | 10 | 80% (10) | 20% (2) | 0% (0) | Yes (Consensus) |
| In case of neoadjuvant treatment with positive lymph nodes on FNA/Core biopsy, lymph node marking BEFORE treatment should be done, and this lymph node should be removed after neoadjuvant treatment | 10 | 80% (10) | 20% (2) | 0% (0) | Yes (Consensus) |
| Cut-off number of Lymph nodes to be marked in the NAC setting is <3 | 10 | 70% (6) | 20% (2) | 10% (1) | Yes (Consensus) |
| My preferences for marking lymph nodes are |  |  |  |  |  |
| Carbon marking | 10 | 10% (1) | 0% (0) | 0% (0) | .. |
| Clip without filler | 10 | 40% (4) | 0% (0) | 0% (0) | .. |
| Clip with filler | 10 | 30% (3) | 0% (0) | 0% (0) | .. |
| Radioactive seed | 10 | 20% (2) | 0% (0) | 0% (0) | .. |
| RFID | 10 | 0% (0) | 0% (0) | 0% (0) | .. |
| Magnetic seed | 10 | 20% (2) | 0% (0) | 0% (0) | .. |
| ROLL | 10 | 0% (0) | 0% (0) | 0% (0) | .. |
| A combination of markers | 10 | 10% (1) | 0% (0) | 0% (0) | .. |
| Other (specify) | 10 | 0% (0) | 0% (0) | 0% (0) | .. |
| -> no consensus found |  |  |  |  |  |
| In the case of palpable lymph nodes on clinical examination, US should always be performed. | 10 | 90% (9) | 10% (0) | 0% (0) | Yes (Consensus) |
| In case of confirmed metastatic lymph nodes, a CT/PET_CT scan must always be performed before treatment | 10 | 60% (6) | 20% (2) | 20% (2) | Yes (Majority) |
| In the case of neoadjuvant treatment with positive lymph nodes, core biopsy of the lymph node is mandatory to provide tumor tissue for adequate therapy-guiding tests | 10 | 70% (7) | 30% (3) | 0% (0) | Yes (Consensus) |

# **Supplemental Table 4. WP 4: Questions pertaining to diagnostic tumor marking of the breast in women with an operable breast cancer with the intent to facilitate breast conservation.**

| Statements | No. of responses | Percentage votes | | | Voting result |
| --- | --- | --- | --- | --- | --- |
|  |  | **Yes (n)** | **No (n)** | **Abstain (n)** |  |
| In all patients with an imaging finding suspicious enough to warrant biopsy (BIRADS-4 and 5), a marker should be placed to facilitate pre-surgical localization | 10 | 30% (3) | 70% (7) | 0% (0) | No (Consensus) |
| In all patients with a potential indication for later image-guided lesion localization: a marker that can be re-localized with ultrasound guidance is preferred | 10 | 90% (9) | 10% (1) | 0% (0) | Yes (Consensus) |
| Any type of tumor localization at diagnosis can be used as long as preoperative localization is feasible | 10 | 80% (8) | 10% (1) | 10% (1) | Yes (Consensus) |
| Preoperative marking should be performed for all non-palpable breast cancers | 10 | 90% (9) | 0% (0) | 10% (1) | Yes (Consensus) |
| Preoperative marking should be performed for all palpable breast cancers | 10 | 10% (1) | 60% (6) | 30% (3) | No (Consensus) |
| The guide wire should be placed in the center of the target lesion in case the lesion is more or less spherical | 10 | 80% (8) | 20% (2) | 0% (0) | Yes (Consensus) |
| The guide wire should not be placed in the center in case the lesion, but in at least two opposing poles of a lesion (bracketing) that is irregular in shape | 10 | 40% (4) | 40% (4) | 20% (2) | No Decision |
| All patients with additional lesions (multifocality, EIC) seen in MRI or any other technique should be discussed at the MDT in order to plan the pre-operative marking strategy | 10 | 100% (10) | 0% (0) | 0% (0) | Yes (Consensus) |
| Direct interaction with the surgeon is important to plan the localization and bracketing of lesions to help the surgeon understand where the guide wires were placed | 10 | 100% (10) | 0% (0) | 0% (0) | Yes (Consensus) |
| All patients with additional lesions (multifocality, EIC) seen in MRI or any other technique should be pre-surgically biopsied | 10 | 80% (8) | 10% (1) | 10% (1) | Yes (Consensus) |
| All patients with additional lesions (multifocality, EIC) seen in MRI or any other technique should be properly bracketed | 10 | 70% (7) | 30% (3) | 0% (0) | Yes (Consensus) |
| It should be possible for any marker that is used for preoperative staging to be re-localized intraoperatively (e.g. sonographic image, magnetic, radioactive, radiofrequency) | 10 | 80% (8) | 0% (0) | 20% (2) | Yes (Consensus) |
| In patients with multicentric lesions and planned breast conservation: each lesion should be marked. | 10 | 80% (9) | 20% (2) | 0% (0) | Yes (Consensus) |
| My preference for marking the primary tumor and additional lesions is |  |  |  |  |  |
| Clip without filler | 10 | 30% (3) | 0% (0) | 0% (0) | .. |
| Clip with filler | 10 | 40% (4) | 0% (0) | 0% (0) | .. |
| Radioactive seed | 10 | 10% (1) | 0% (0) | 0% (0) | .. |
| RFID | 10 | 10% (1) | 0% (0) | 0% (0) | .. |
| Magnetic seed | 10 | 10% (1) | 0% (0) | 0% (0) | .. |
| ROLL | 10 | 10% (2) | 0% (0) | 0% (0) | .. |
| A combination of markers | 10 | 0% (0) | 0% (0) | 0% (0) | .. |
| Other (guidewire) | 10 | 10% (1) | 0% (0) | 0% (0) | .. |
| -> no consensus found |  |  |  |  |  |
| The orientation protocol used in surgical specimens has to be known by the radiologist and the pathologist | 10 | 100% (10) | 0% (0) | 0% (0) | Yes (Consensus) |
| Removal of localizing marker has to be documented with surgical specimen radiograph | 10 | 100% (10) | 0% (0) | 0% (0) | Yes (Consensus) |

# **Supplemental Table 5. WP 5: Questions pertaining to intraoperative imaging of the specimen.**

| Statements | No. of responses | Percentage votes | | | Voting result |
| --- | --- | --- | --- | --- | --- |
|  |  | **Yes (n)** | **No (n)** | **Abstain (n)** |  |
| Patients with complex lesions (i.e. not unifocal) that have been marked should be discussed (radiologist and surgeon) pre-operatively | 10 | 100% (11) | 0% (0) | 0% (0) | Yes (Consensus) |
| In patients with calcifications and/or pre-operatively placed markers, a specimen radiography should be performed in order to confirm the removal | 10 | 100% (11) | 0% (0) | 0% (0) | Yes (Consensus) |
| Regardless of the location of the X-ray source for the specimen radiograph (Radiology department, OR or Pathology department), both the radiologist and the surgeon have to evaluate the specimen image | 10 | 90% (9) | 10% (1) | 0% (0) | Yes Consensus) |
| Radiologist and surgeon should be able to communicate real time while evaluating intraoperative x-ray images | 10 | 90% (9) | 10% (1) | 0% (0) | Yes Consensus) |
| For better accuracy in special orientation, the specimen should be placed on its support by the surgeon | 10 | 100% (11) | 0% (0) | 0% (0) | Yes (Consensus) |
| Multimodal specimen evaluation (US+X-ray) should always be performed in the OR and guide, in selected cases, the indication for targeted frozen section on margins | 10 | 50% (5) | 50% (5) | 0% (0) | No Decision |
| When taking advantage of intraoperative US, this should be performed both before beginning the surgical procedure, to plan the resection, and after for gross evaluation of surgical margins | 10 | 80% (8) | 0% (0) | 20% (2) | Yes (Consensus) |
| Intraoperative ultrasound of the specimen is only valid to locate the clip/marker, not for margin evaluation | 10 | 90% (10) | 0% (0) | 10% (1) | Yes (Consensus) |
| In case of a missing marker in the specimen as proven by X-ray or US, the surgeon has to be informed immediately | 10 | 100% (10) | 0% (0) | 0% (0) | Yes (Consensus) |

# **Supplemental Table 6. WP 6: Questions pertaining to the radiological-pathological correlation of breast cancer cases.**

| Statements | No. of responses | Percentage of votes | | | Voting result |
| --- | --- | --- | --- | --- | --- |
|  |  | **Yes (n)** | **No (n)** | **Abstain (n)** |  |
| Tumor size, disease extent, lesion focality and heterogeneity are essential parameters that characterize breast carcinomas and should be reported as assessed with multimodality imaging methods preoperatively and with detailed postoperative histopathological work-up of the specimen | 10 | 100% (10) | 0% (0) | 0% (0) | Yes (Consensus) |
| The radiological assessment results of these parameters must be correlated to the results of histopathological assessment in order to reach a definite interdisciplinary consensus diagnosis | 10 | 100% (10) | 0% (0) | 0% (0) | Yes (Consensus) |
| Specimen radiographs (of the intact specimen, the sliced specimen, or both) facilitate this correlation by guiding the pathologist in sampling the tissue for histological examination and should be provided in all cases in which simple macroscopic examination may result in under-sampling of the specimen | 10 | 100% (10) | 0% (0) | 0% (0) | Yes (Consensus) |
| Large-format histopathology has clear advantages over conventional sampling techniques in determining these parameters and should be preferably used | 10 | 90% (10) | 0% (0) | 10% (1) | Yes (Consensus) |

# **Supplemental Table 7. WP 7: Questions pertaining to benchmark timelines for imaging studies during staging.**

| Statements | No. of responses | Percentage of votes | | | Voting result |
| --- | --- | --- | --- | --- | --- |
|  |  | **Yes (n)** | **No (n)** | **Abstain (n)** |  |
| Most quality-certification requirements for Breast cancer (BC) management define timeline limits for diagnosis and treatment of newly diagnosed BC patients. According to timeline benchmarks, all efforts should be done to have a comprehensive baseline assessment finished to commence treatment within 4 weeks from diagnosis. Do you think this general timeline indication is reasonable? | 10 | 90% (10) | 10% (1) | 0% (0) | Yes (Consensus) |
| All quality certification bodies agree upon the fact that diagnosis should be given to the patient as soon as possible. Some require the diagnosis to be delivered within 5 working days from first visual contact with the patient, even if this entails giving only partial information limited to the nature of the lesion (benign/malignant) | 10 | 60% (7) | 40% (4) | 0% (0) | Yes (Majority) |
| Do you think a limited diagnosis, which does not allow discussing treatment details, is beneficial for the patient? | 10 | 30% (3) | 70% (7) | 0% (0) | No (Consensus) |
| Do you think that waiting for a second consultation to discuss treatment options, while aware of the tumor diagnosis, increases the level of anxiety for the patient? | 10 | 80% (8) | 20% (2) | 0% (0) | Yes (Consensus) |
| Do you think that a waiting time of 7 working days is reasonable to give complete diagnosis and discuss tailored treatment options? | 10 | 90% (10) | 10% (1) | 0% (0) | Yes (Consensus) |
| In some countries, it is required to allow 24h between patient’s informed consent and performing a Breast biopsy. In order to keep waiting time as short as possible, do you think that triple assessment (examination + imaging + biopsy) should be performed during first Clinical consultation without further delay? | 10 | 90% (10) | 10% (1) | 0% (0) | Yes (Consensus) |
| With the increasing use of preoperative MRI staging, we are facing frequent delays to treatment due to indications for further diagnostic assessment of suspicious findings on MRI. Given precise indications for the use of preoperative MRI, do you think that the procedure should be booked as early as first consultation, when those indications apply? | 10 | 100% (10) | 0% (0) | 0% (0) | Yes (Consensus) |
